# Non-Invasive Physical Plasma Reduces the Inflammatory Response in Microbially Prestimulated Human Gingival Fibroblasts

**DOI:** 10.3390/ijms242216156

**Published:** 2023-11-10

**Authors:** Benedikt Eggers, Matthias Bernhard Stope, Jana Marciniak, Alexander Mustea, Sigrun Eick, James Deschner, Marjan Nokhbehsaim, Franz-Josef Kramer

**Affiliations:** 1 Department of Oral, Maxillofacial and Plastic Surgery, University Hospital Bonn, 53111 Bonn, Germany; franz-josef.kramer@ukbonn.de; 2 Department of Gynecology and Gynecological Oncology, University Hospital Bonn, 53127 Bonn, Germany; matthias.stope@ukbonn.de (M.B.S.); alexander.mustea@ukbonn.de (A.M.); 3 Department of Orthodontics, University Hospital Bonn, 53111 Bonn, Germany; jana.marciniak@ukbonn.de; 4 Department of Periodontology, School of Dental Medicine, University of Bern, 3010 Bern, Switzerland; sigrun.eick@unibe.ch; 5 Department of Periodontology and Operative Dentistry, University Medical Center of the Johannes Gutenberg University, 55131 Mainz, Germany; james.deschner@uni-mainz.de; 6 Section of Experimental Dento-Maxillo-Facial Medicine, University Hospital Bonn, 53111 Bonn, Germany; m.saim@uni-bonn.de

**Keywords:** non-invasive physical plasma, cold plasma, non-thermal plasma, antimicrobial effect, inflammation, gingival fibroblasts, dentistry, periodontology

## Abstract

Non-invasive physical plasma (NIPP), an electrically conductive gas, is playing an increasingly important role in medicine due to its antimicrobial and regenerative properties. However, NIPP is not yet well established in dentistry, although it has promising potential, especially for periodontological applications. The aim of the present study was to investigate the effect of NIPP on a commercially available human gingival fibroblast (HGF) cell line and primary HGFs in the presence of periodontitis-associated bacteria. First, primary HGFs from eight patients were characterised by immunofluorescence, and cell numbers were examined by an automatic cell counter over 5 days. Then, HGFs that were preincubated with *Fusobacterium nucleatum* (*F.n.*) were treated with NIPP. Afterwards, the IL-6 and IL-8 levels in the cell supernatants were determined by ELISA. In HGFs, *F.n.* caused a significant increase in IL-6 and IL-8, and this *F.n.*-induced upregulation of both cytokines was counteracted by NIPP, suggesting a beneficial effect of physical plasma on periodontal cells in a microbial environment. The application of NIPP in periodontal therapy could therefore represent a novel and promising strategy and deserves further investigation.

## 1. Introduction

Periodontitis is a chronic inflammatory disease of the oral cavity characterised by the destruction of the surrounding tissues of a tooth. The underlying mechanism of this disease is complex, but immunological inflammatory reactions to various oral bacteria, such as *Porphyromonas gingivalis (P.g.)*, *Fusobacterium nucleatum (F.n.)*, or *Aggregatibacter actinomycetemcomitans*, play a crucial role, leading to the degradation of soft and hard tissues [1]. During the progression of the disease, a soft tissue pocket develops around the tooth, providing a suitable anaerobic environment for the microbial plaque [2]. Left untreated, the bacterial infection increases many times over, so that in the long term the bone tissue is resorbed, and teeth can be lost [3,4,5].

In particular, the Gram-negative, obligate anaerobic pathogen *F.n.* plays an important role in periodontitis. It serves as a supportive bridging organism that is able to link obligate anaerobic and aerobic pathogens and helps to develop biofilms [6]: thus, *F.n.* indirectly enhances the creation of the environments in which *P.g.* and many obligate anaerobes can survive during dental plaque maturation [7,8]. The immune response of the host to the periodontopathogens leads to the production of a number of inflammatory mediators, matrix-degrading enzymes, and osteoclastic markers [6].

The regular treatment of periodontitis is performed by subgingival instrumentation with hand instruments and/or machine-driven devices [9,10]. This therapy can be supported with CHX-containing mouth rinses, gels, or even chips that are inserted into the sulcus of the affected teeth [11,12]. The main goal of the therapy is to reduce pathogens and thus inflammation [9,10]. However, when the biofilm is removed, the periodontal cells repopulate the wound. Usually a long-junctional epithelium as an interface between the gingiva and the root is formed [13]. However, ideally, periodontal therapy also leads to a restoration of the original form, structure, and function of the tissues lost due to periodontitis. However, this is usually only possible with regenerative periodontal procedures [14,15]. In severe and rapidly progressive cases of periodontitis, the use of antibiotics as an adjunct to the non-surgical treatment of periodontitis can be considered. In order to avoid further development of antibiotic resistance, alternative methods are the subject of current research [16]. Non-invasive physical plasma (NIPP) has been described as an excited gas state. Its biomedical effects are mainly mediated by reactive oxygen and nitrogen species formed at the interface between the NIPP and the ambient atmosphere by energy transfer [17]. NIPP promotes tissue regeneration including wound healing and exerts antimicrobial effects on pathogens [18,19,20,21]. In vitro studies have shown that NIPP can be used not only to eliminate individual oral pathogens such as *F.n.* or *P.g.* but also to eliminate oral biofilms [22,23,24]. NIPP is also used for the decontamination and sterilisation of implants [25].

So far, little is known about the regulatory effect of NIPP on oral cells. In a previous study, we have demonstrated that NIPP promotes regeneration-associated cell functions in gingival fibroblasts and keratinocytes [26]. In addition, NIPP application to gingival cells also downregulated the synthesis of proinflammatory molecules [27]. Therefore, the aim of the present study was to investigate the effect of NIPP on a commercially available human gingival fibroblast (HGF) cell line and primary HGFs in the presence of periodontitis-associated bacteria. It was hypothesised that NIPP would exert anti-inflammatory effects on gingival cells in the presence of periodontitis-associated bacteria.

## 2. Results

**Figure 1:**
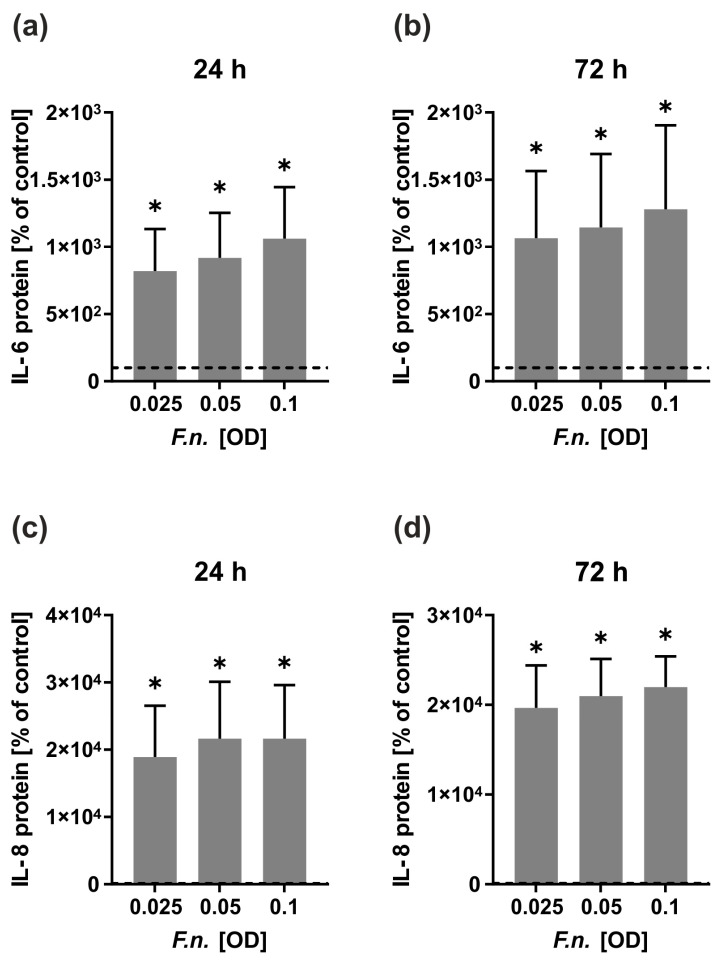
Effect of different bacterial concentrations on IL-6 and IL-8 protein levels in the supernatants of a HGF cell line (*n* = 3). (**a**) IL-6 protein levels at 24 h; (**b**) IL-6 protein levels at 72 h; (**c**) IL-8 protein levels at 24 h; (**d**) IL-8 protein levels at 72 h. Controls were set to 100% (dashed line). * Significantly different from control (*p* < 0.05).

Since the aim of our study was to investigate the effect on microbial infection, the first step was to determine the optimal concentration of the bacteria for the prestimulation of the cells in the following experiments. All used bacterial concentrations (OD: 0.025–0.1) caused a significant increase in the IL-6 and IL-8 protein levels in the HGF cell line (*p* < 0.05) (Figure 1a–d). As the stimulatory effect of the different concentrations was quite similar, the lowest bacterial concentration (OD: 0.025) was used to simulate a microbial environment in the subsequent experiments.

**Figure 2:**
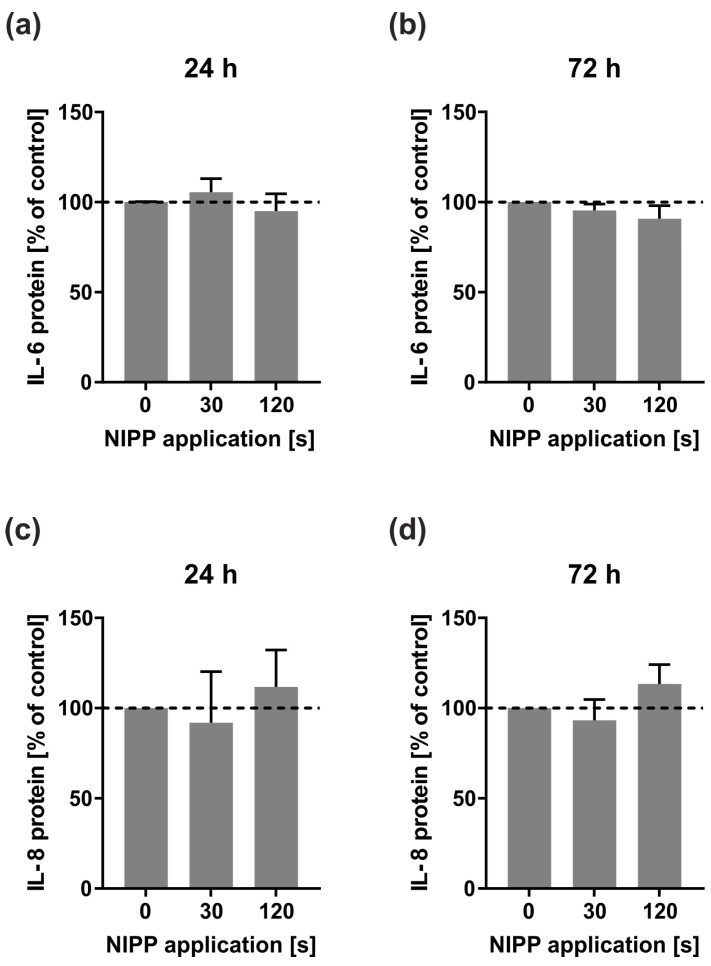
Effect of different NIPP application times on IL-6 and IL-8 protein levels in the supernatants of a HGF cell line (*n* = 3). (**a**) IL-6 protein levels at 24 h; (**b**) IL-6 protein levels at 72 h; (**c**) IL-8 protein levels at 24 h; (**d**) IL-8 protein levels at 72 h. Controls were set to 100% (dashed line).

Then, we sought to determine whether NIPP alone would have an effect on the IL-6 and IL-8 protein levels in the supernatants in the HGF cell line. NIPP was applied for 30 s or 120 s. As shown in Figure 2, NIPP did not alter the IL-6 and IL-8 levels in the supernatants of HGF at 24 h and 72 h. For the following experiments, in which the effects of NIPP on bacterial-stimulated HGF cells were examined, a NIPP application time of 30 s was used.

**Figure 3:**
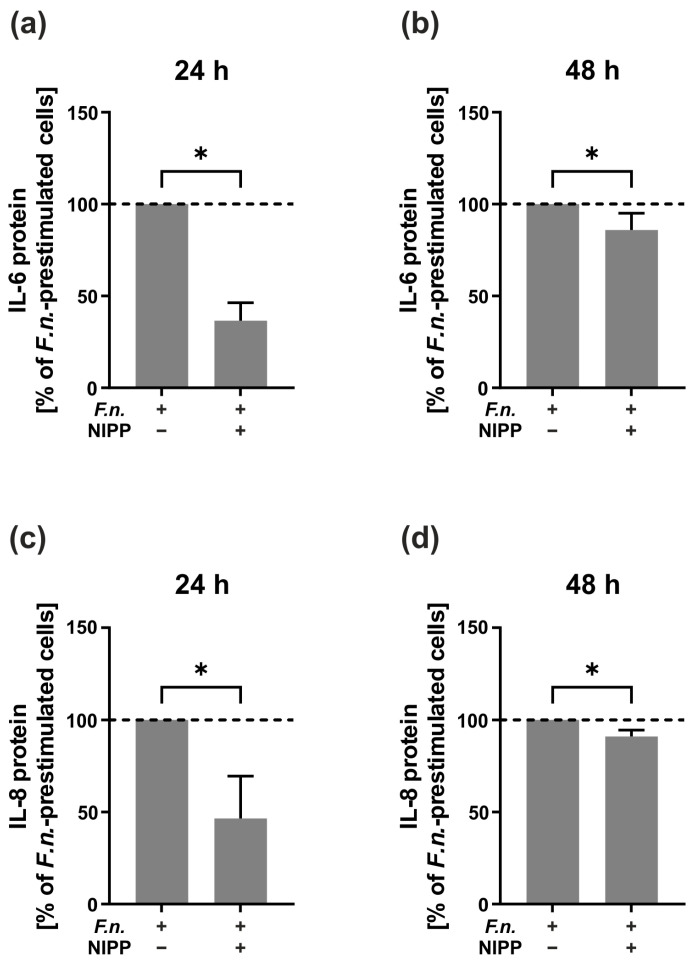
Effects of NIPP on IL-6 and IL-8 protein levels in the supernatants of the *F.n.*-prestimulated HGF cell line (*n* = 4). (**a**) IL-6 protein levels at 24 h; (**b**) IL-6 protein levels at 48 h; (**c**) IL-8 protein levels at 24 h; (**d**) IL-8 protein levels at 48 h. *F.n.*-prestimulated cells were set to 100% (dashed line). * Significantly different (*p* < 0.05).

Next, we investigated the modulatory effect of NIPP on the HGF cell line preincubated with *F.n.* As shown in Figure 3, the IL-6 and IL-8 protein levels were significantly reduced at 24 h and 48 h (*p* < 0.05) (Figure 3a–d). The inhibitory effect of NIPP on the IL-6 and IL-8 protein levels of the *F.n.*-prestimulated HGF cells was more pronounced at 24 h as compared to 48 h (Figure 3a–d).

**Figure 4:**
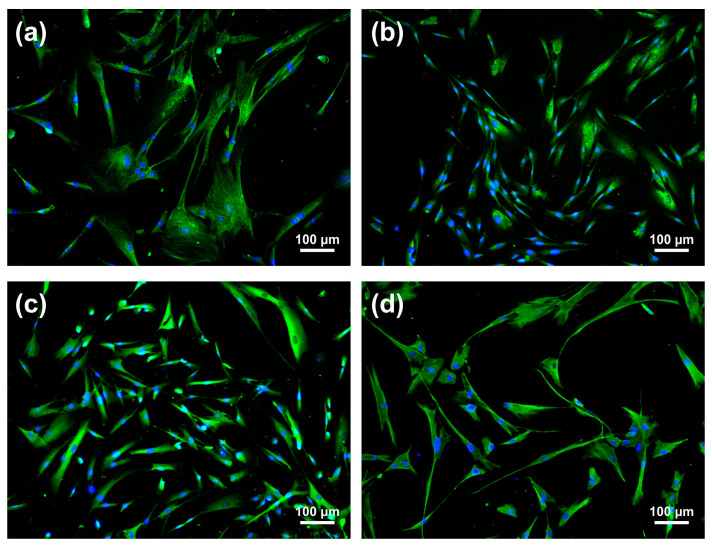
Characterisation of primary gingival cells by immunofluorescence for fibroblast markers. (**a**) *Alpha SMA staining*; (**b**) Collagen 1 staining; (**c**) S100A4 staining; (**d**) Vimentin staining. Representative images of primary HGF cells are shown. Scale bar represents 100 µm.

Next, it was examined whether the antimicrobial effects of NIPP on the *F.n.*-prestimulated HGF cell line were also found on primary HGF cells. HGF cells from eight patients were isolated, cultured, and subsequently characterised. All cells demonstrated a spindle-shaped morphology and expressed alpha-SMA (Figure 4a), collagen 1 (Figure 4b), S100A4 (Figure 4c), and Vimentin (Figure 4d), indicating a fibroblast-like phenotype.

**Figure 5:**
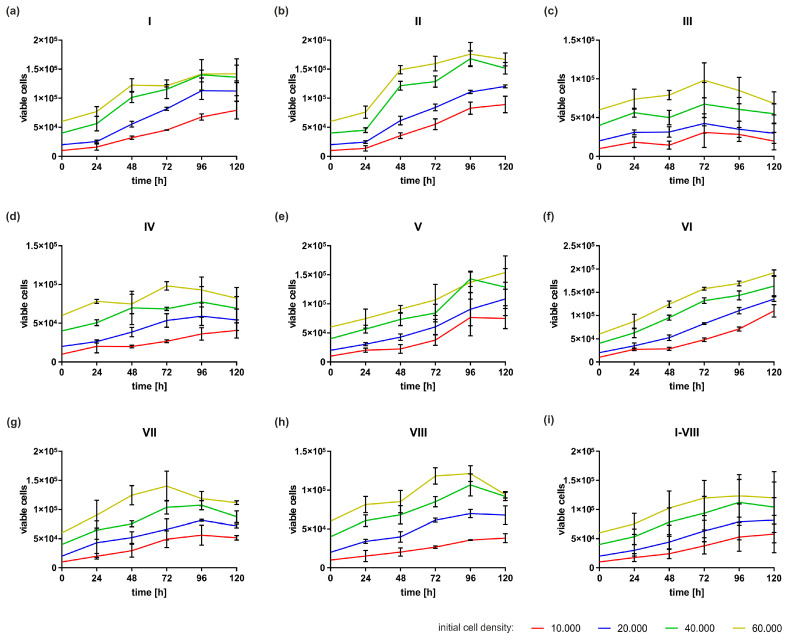
Growth kinetics of primary HGF cells. Cells seeded at different densities were analysed using a CASY Cell Counter and Analyzer Modell TT over a period of 120 h. (**a**–**h**) HGFs from eight different patients; each subfigure represents findings from one patient (*n* = 3); (**i**) Growth kinetics of HGFs for all donors together (*n* = 24).

Subsequently, the growth characteristics of the primary cells from the eight donors were analysed (Figure 5a–i). The cells seeded at different densities remained viable over the 120 h observation time and increased in number, as shown in Figure 5a–i, demonstrating that all eight cell populations were eligible for further investigation. Since exponential growth was observed in all cell donors at the initial cell densities of 20,000 and 40,000 (Figure 5i), it was decided to seed primary cells at a density of 40,000 in the following experiments.

**Figure 6:**
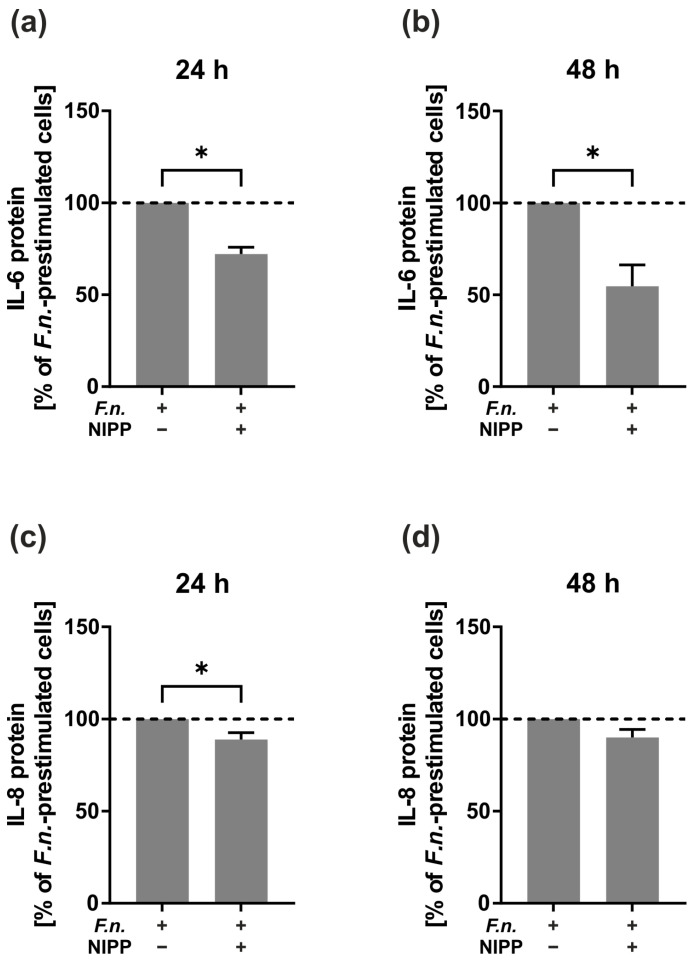
Effects of NIPP on IL-6 and IL-8 protein levels in the supernatants of the *F.n.*-prestimulated primary HGF cells (*n* = 8). (**a**) IL-6 protein levels at 24 h; (**b**) IL-6 protein levels at 48 h; (**c**) IL-8 protein levels at 24 h; (**d**) IL-8 protein levels at 48 h. *F.n.*-prestimulated cells were normalised to 100% (dashed line). * Significantly different (*p* < 0.05).

Finally, we studied the regulatory effect of NIPP on the primary HGF cells preincubated with *F.n.* As analysed by ELISA, the IL-6 and IL-8 protein levels in the supernatants were also significantly increased in the primary HGF cells after exposure to *F.n.* As compared to the control, the IL-6 level was elevated after *F.n.* treatment (*p* < 0.05) by 2.03 × 10^2^-fold at 24 h and 1.49 × 10^3^-fold at 48 h. Similarly, the exposure of cells to *F.n.* caused elevated IL-8 levels (*p* < 0.05) by 2.68 × 10^2^-fold at 24 h and by 3.59 × 10^2^-fold at 48 h, in comparison with the untreated control. When the *F.n.*-pretreated cells were subjected to NIPP, the IL-6 protein levels were significantly decreased at 24 h and 48 h (*p* < 0.05) (Figure 6a,b) and the IL-8 protein levels at 24 h (*p* < 0.05) (Figure 6c). As observed for the HGF cell line, NIPP alone had no remarkable effect on the primary HGF cells.

## 3. Discussion

**Figure 7:**
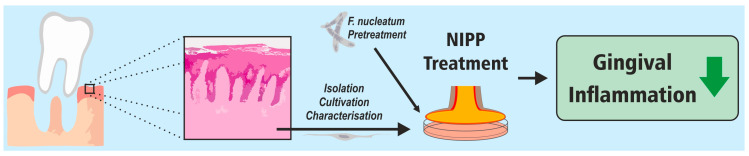
Effects of NIPP on microbially prestimulated primary HGF.

In the present study, we investigated the effect of NIPP on the regulation of the inflammatory cytokines IL-6 and IL-8 in microbially prestimulated gingival fibroblasts. In our study, NIPP inhibited the microbially induced protein levels of these inflammatory mediators in both the HGF cell line and the patient-derived primary HGF cells, suggesting that NIPP can counteract the proinflammatory effects of *F.n.* on periodontal cells (Figure 7).

The antimicrobial efficacy of NIPP is well known [28]. Many authors have described the direct antimicrobial effect of NIPP against oral pathogens, such as *P.g.*, *Streptococcus mutans*, *Candida albicans*, *Prevotella intermedia*, or *F.n.* [23,29,30]. Moreover, it is an accepted fact that infection with these bacteria leads to a strong cytokine response of the infected cells as well as the unaffected surrounding cells [31,32]. Previously, we have investigated the effect of NIPP on various cytokines in human gingival fibroblasts; notably, IL-6 and IL-8 have been strongly downregulated by NIPP [27], which is why we focused on these cytokines in the present study.

Periodontitis is a chronic inflammatory disease characterised by progressive degradation of the periodontal ligament, alveolar bone, and gingiva [1]. Various molecules that are released during the progression of the disease are responsible for the destruction of this tissue [33,34]. Additionally, elevated levels of IL-6 and IL-8 secretion have been observed in patients suffering from periodontitis [35]. These cytokines IL-6 and IL-8 are proinflammatory cytokines, mainly released during acute phase reactions. IL-6 contributes to lymphocyte differentiation in the early inflammatory phase and stimulates chemotaxis of other immune cells [36,37]. This cytokine plays an important role in chronic inflammatory and autoimmune diseases [38]. IL-8 also supports chemotaxis [39]. Both cytokines also play an important role in wound healing, e.g., through the release of growth factors [40,41].

In the present study, it was observed that the IL-6 protein levels in supernatants were increased by *F.n.* and that the microbially induced elevation of this inflammatory mediator was reduced by NIPP. The anti-inflammatory effect of NIPP in reducing IL-6 protein levels has also been demonstrated by other authors in patients with peri-implantitis, although the authors did not differentiate between the individual bacteria [42]. Interestingly, in the previous study, we even observed a downregulation of IL-6 in unstimulated HGF cells [27]. In the present study, NIPP had no significant effect on IL-6 regulation in HGF cells. However, different NIPP devices were used in both studies: in the present study, we used a dielectric barrier discharge (DBD) NIPP device in contrast to the plasma jet described previously [27]. Thus, a plasma jet seems to have a stronger influence on IL-6 secretion than a DBD device. Differences in the effect of the various NIPP devices have also been described by other authors; for example, differences in the direct antimicrobial effect on different pathogens have been shown between a DBD and plasma jet and between different DBDs [43,44].

Interestingly, it is not only the downregulating effects of NIPP on cytokine production that have been described in the literature; the stimulatory action of NIPP on IL-6 has also been shown in prostate carcinoma cells [45]. Thus, it seems that different cells react differently to NIPP. This could be comparable to the different effects of NIPP on cancer cells, which induce apoptosis rather than regeneration-associated effects [46,47]. However, further studies are needed to clarify such observations.

Furthermore, NIPP exerted similar effects on IL-8 in the HGF cells stimulated with *F.n.*, as observed for IL-6. The anti-inflammatory effect of NIPP on IL-8 has already been demonstrated in clinical studies on the inflammatory phase of diabetic foot ulcers: the authors observed a strong decrease in cytokines such as IL-1, IL-8, INF-γ, and TNFα. [48]. Our results also confirm these findings in HGF cells. Interestingly, as with IL-6, treatment with NIPP had no effect on the regulation of IL-8 in unstimulated HGF cells—both in the cell line and in primary cells. The difference from the previously published study [27] could also be due to the use of different NIPP devices and should be further investigated.

The biostimulatory effect of NIPP is particularly due to reactive oxygen and nitrogen species, which represent essential elements for various signalling molecules and control; for example, vasodilatation, collagen synthesis, or immune modulation [49,50]. The results of this study, as well as the results of previous studies [26,51,52], underline that these antimicrobial and regenerative effects can also be observed in intraoral cells.

In a clinical study, Küçük et al. showed that the use of NIPP in the treatment of periodontitis also led to a reduction in putative periodontopathogens, reduced their recolonization, and provided additional attachment gain [53]. Additionally, a clinical study has already demonstrated a positive effect of NIPP on intraoral wound healing [54]. Therefore, the present study may partially elucidate the background of the underlying clinical mechanisms.

In addition, other methods have also been described in the literature that can decrease cytokine levels in gingival cells: Papadelli et al. have shown that both 810 nm diode and Nd:YAG 1064 nm lasers can decrease the production of IL-6 and IL-8 in response to LPS in gingival fibroblasts [55]. Additionally, a special peptide inhibitor has been described that reduces the virulence of *P.g.* [56]. Moreover, antibiotics such as doxycycline have an effect on reducing IL-6 in gingival cervical fluid samples from patients with chronic periodontitis [57]. However, in consideration of the rise of antibiotic resistance in hospitals, communities, and the environment [58], the use of NIPP for inflammatory diseases would be a preferable therapeutic alternative.

With regard to a possible clinical application of NIPP, the advantages of the different NIPP application types would also need to be discussed. A DBD device allows the use of differently sized probes but only works at a certain distance from the tissue to be treated [59]. A plasma jet creates an effluent that can easily be used to treat different shaped tissues; however, the narrow diameter of the effluent makes it difficult to work on large areas [60]. Since the oral cavity is not a flat surface and treatment is often limited to small areas, the use of a plasma jet seems more advantageous. However, only the Plasma One device we used in this study is licensed for clinical intraoral use.

However, this study also has limitations. On the one hand, we only focused on the cytokines IL-6 and IL-8. Other cytokines, such as IL-1, INF-γ, or TNFα, also play an important role in inflammatory processes and should be investigated in further studies. On the other hand, further studies should not only focus on anti-inflammatory cytokines but also on pathways and receptors in order to understand the effects of NIPP. Furthermore, in the present study, gingival fibroblasts were incubated only with *F.n.* As periodontitis is a polymicrobial disease [61,62], the interactions of NIPP with other periodontal pathogens would also be of interest. It is well known that *P.g.* as a keystone pathogen plays a critical role in the development and progression of periodontitis [63] and should therefore be the subject of further studies. Nevertheless, according to the keystone-pathogen hypothesis, it is clear that the entire biofilm more or less is involved in the development and progression of periodontitis [64]; so, future experiments should also be carried out with other microorganisms or entire biofilms. However, we focused on the effects of *F.n.*, since this microorganism is an important bridging bacterium between early and late biofilm colonisers. Moreover, *F.n.* plays an important role in both gingivitis and periodontitis. In the present study, *F.n.* was used as an inactivated lysate. Further studies should clarify whether viable *F.n.* would exert similar, different, or additional effects on periodontal cells. It should also be considered that this lysate contains a variety of virulence factors, such as lipopolysaccharides. Further studies could also investigate the effects of the single virulence factors of *F.n.* on the cells. However, this method has already been described in many in vitro studies [31]. In addition, we have focused only on HGF cells. It should also be investigated whether NIPP also counteracts the effect of *F.n.* or other bacteria on keratinocytes or periodontal ligament cells. Overall, we focused on a single NIPP treatment with a duration of 30 s. As there are no known negative effects associated with repeated NIPP treatments [65,66], it should also be investigated whether repeated applications can enhance the anti-inflammatory effect on gingival cells. Finally, it must be taken into account that this study is an in vitro study, which allows only limited conclusions to be drawn about the therapy of inflammatory diseases with NIPP.

On the other hand, our study also has numerous strengths; for example, the antimicrobial effect of NIPP was not only demonstrated in one cell line but was also confirmed in primary cells from eight donors. In addition, the regulatory effect of NIPP on two classic established inflammatory mediators, which have been shown to be causally associated with periodontitis, was investigated. Furthermore, the analyses for these inflammatory mediators were not carried out at the expression but at the protein level.

In summary, in the present in vitro study, we examined the actions of NIPP on IL-6 and IL-8 in the supernatants of microbially prestimulated gingival fibroblasts. NIPP counteracted the microbially induced protein levels of both inflammatory mediators, suggesting that NIPP can exert anti-inflammatory effects on periodontal cells in a microbial environment.

## 4. Materials and Methods

### 4.1. Cell Isolation and Cell Culture

Primary HGF cells were anonymously derived by explant cultures from gingival biopsies discarded during routine surgery from 8 donors at the Department of Oral, Maxillofacial, and Plastic Surgery at the University Hospital Bonn according to the protocol of Abreu et al. [67]. The study was approved by the Ethics Committee of the University Hospital of Bonn (#111/17). Cells were propagated in 75 cm^2^ flasks (Greiner bio-one, Frickenhausen, Germany) in Dulbecco’s modified essential medium (DMEM; Invitrogen, Waltham, MA, USA), supplemented with 10% fetal bovine serum (FBS; Invitrogen) and 1% penicillin/streptomycin (Invitrogen), and maintained in a humidified incubator at 37 °C and 5% CO_2_. The culture medium was changed every 2–3 d. The commercially available cell line HGF-1 (CRL-2014; ATCC (Manassas, VA, USA) was used as the positive control. Experiments were performed in passages 3–5.

### 4.2. Characterisation of Primary Gingival Fibroblasts

Primary HGF cells at passage 3 were seeded on coverslips (Thermo Fisher Scientific, Waltham, MA, USA) and cultivated as described above. For immunohistochemistry, cells were fixated using 4% paraformaldehyde (Sigma-Aldrich, Saint-Louis, MO, USA) for 10 min and 0.05% Triton^®^ X-100 (Sigma-Aldrich) for 5 min. For characterisation, the fixated cells were incubated with primary antibodies mouse anti-alpha smooth muscle Actin (1:800; abcam, Tokyo, Japan), rabbit anti-Collagen I (1:400; abcam), rabbit anti-S100A4 (1:1000; abcam), or rabbit anti-Vimentin (1:1000; abcam) in 1% BSA in a humid chamber at 4 °C overnight. Goat Anti-Rabbit IgG H&L Alexa Fluor 488 (1:800; abcam) or Goat Anti-Mouse IgG H&L Alexa Fluor 488 (1:800; abcam), respectively incubated at room temperature for 60 min, were used as secondary antibodies. Stained cells were analysed using the ZOE Fluorescent Cell Imager (Bio-Rad, Hercules, CA, USA).

### 4.3. Growth Kinetics

For the cell proliferation analysis, the cells were seeded in 24-well cell culture plates (Greiner bio-one) at different densities. The adherent cells were detached with 0.1% trypsin/0.04% EDTA (Sigma-Aldrich) and resuspended in CASY-ton solution (Roche Applied Science). The cells were analysed using the CASY Cell Counter and Analyzer model TT (Roche Applied Science, Mannheim, Germany) with a capillary of 150 µm diameter. The number of living cells was determined.

### 4.4. Microbial Stimulation and NIPP Application

**Figure 8:**
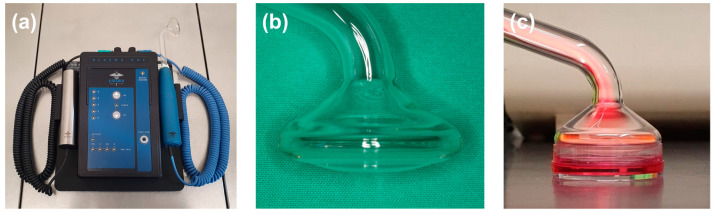
The NIPP device used for the experiment was the DBD device Plasma ONE (Plasma MEDICAL SYSTEMS^®^, Nassau, Germany). (**a**) Operation unit; (**b**) patient probe PS30; (**c**) NIPP treatment of HGF cells.

The cells were seeded in 35 mm Petri dishes (Greiner bio-one) at a density of 40,000. One day prior to the experiments, the FBS concentration was reduced to 1%. The cells were preincubated with lysates of inactivated *Fusobacterium nucleatum* (ATCC 25586) to simulate periodontal inflammation. To inactivate the bacteria, it was suspended in phosphate-buffered saline (OD_660_ = 1.0, corresponding to 1.2 × 10^9^ bacterial cells/mL) and sonicated twice (160 W for 15 min). After 24 h, the cells were directly treated with NIPP (Plasma ONE, Plasma MEDICAL SYSTEMS^®^, Nassau, Germany) for 30 s with an output of 18 kV (Figure 8a–c). The treatment of the medium-covered HGF cells was conducted at a distance of 2 mm (tip of the probe to the surface of the medium) (Figure 8c). The untreated cells served as a negative control.

### 4.5. Analysis of Protein Levels

The protein levels of IL-6 and IL-8 were analysed in cell culture supernatant after 24 h and 48 h using specific ELISA kits (both Bio-Techne; Minneapolis, MN, USA) according to the manufacturer’s instructions. The optical density was measured at 450 nm using a microplate reader (Epoch™ Microplate Spectrophotometer, BioTek Instruments, Winooski, VT, USA). In addition, the total protein concentration was analysed using the Pierce BCA Protein Assay Kit (23227, Thermo Scientific, Pierce Biotechnology, Rockford, IL, USA) and measured at 570 nm. The protein concentrations of IL-6 and IL-8 were normalised to the total protein concentration.

### 4.6. Statistical Analysis

The statistical analysis was performed using GraphPad Prism version 10 software (GraphPad Software, Inc., La Jolla, CA, USA) by applying nonparametric tests (Kruskal–Wallis test with post hoc Dunn’s multiple comparisons test, Mann–Whitney U test, and Wilcoxon matched-pairs signed rank test). *p* values below 0.05 were defined as statistically significant.

## 5. Conclusions

In conclusion, to the best of our knowledge, this study is the first to show that NIPP has an anti-inflammatory effect on primary human gingival fibroblasts in an infected environment. Together with the previously demonstrated antibacterial and regenerative effects of NIPP, the clinical application of physical plasma could have promising effects in periodontal therapy, which should be further investigated in clinical trials.

## Data Availability

Not applicable.
